# Hypofractionated stereotactic re-irradiation: treatment option in recurrent malignant glioma

**DOI:** 10.1186/1471-2407-5-55

**Published:** 2005-05-30

**Authors:** Dirk Vordermark, Oliver Kölbl, Klemens Ruprecht, Giles H Vince, Klaus Bratengeier, Michael Flentje

**Affiliations:** 1 Dept. of Radiation Oncology, University of Würzburg, Germany; 2 Dept. of Neurology, University of Würzburg, Germany; 3 Dept. of Neurosurgery, University of Würzburg, Germany

## Abstract

**Background:**

Hypofractionated stereotactic radiotherapy (HFSRT) is one salvage treatment option in previously irradiated patients with recurrent malignant glioma. We analyzed the results of HFSRT and prognostic factors in a single-institution series.

**Methods:**

Between 1997 and 2003, 19 patients with recurrent malignant glioma (14 glioblastoma on most recent histology, 5 anaplastic astrocytoma) were treated with HFSRT. The median interval from post-operative radiotherapy to HFSRT was 19 (range 3–116) months, the median daily single dose 5 (4–10) Gy, the median total dose 30 (20–30) Gy and the median planning target volume 15 (4–70) ml.

**Results:**

The median overall survival (OS) was 9.3 (1.9-77.6+) months from the time of HFSRT, 15.4 months for grade III and 7.9 months for grade IV tumors (p = 0.029, log-rank test). Two patients were alive at 34.6 and 77.6 months. OS was longer after a total dose of 30 Gy (11.1 months) than after total doses of <30 Gy (7.4 months; p = 0.051). Of five (26%) reoperations, none was performed for presumed or histologically predominant radiation necrosis. Median time to tumor progression after HFSRT on imaging was 4.9 months (1.3 to 37.3) months.

**Conclusion:**

HFSRT with conservative total doses of no more than 30 Gy is safe and leads to similar OS times as more aggressive treatment schemes. In individual patients, HFSRT in combination with other salvage treatment modalities, was associated with long-term survival.

## Background

Despite intensive multi-modality treatment including tumor resection, post-operative radiotherapy and frequently adjuvant chemotherapy, the prognosis of malignant glioma continues to be poor. Local recurrence, occuring almost exclusively inside the high-dose volume of post-operative radiotherapy [1], represents a major therapeutic challenge. Re-irradiation as a salvage treatment option is limited by the radiation tolerance of surrounding normal brain tissue. In recent years, single-dose radiosurgery, normofractionated and hypofractionated stereotactic radiotherapy have been investigated as a single modality or in combination with chemotherapy [2-8]. Re-irradiation appeared to be associated with acceptable toxicity when certain treatment volume and dose limits were respected. Median survival in these series was between 7 and 13 months from the time of salvage radiotherapy suggesting a therapeutic benefit in selected patient groups.

Since published data on hypofractionated stereotactic radiotherapy as a sole modality in recurrent malignant glioma are limited [4,5,8], we now analyzed the results of such treatment in 19 consecutive patients treated at a single institution.

## Methods

### Patients

Between 1997 and 2003, 19 patients were treated with hypofractionated stereotactic radiotherapy (HFSRT) for recurrent malignant glioma at the University of Würzburg, Germany. The patient characteristics are summarized in Table 1. All patients had received previous involved-field radiotherapy with 45 to 61 Gy and 94% of patients had been pretreated with chemotherapy, most often nimustine (ACNU) and teniposide, a combination protocol favored by the Neuro-Oncology Working Group of the German Cancer Society [9].

### Treatment technique

HFSRT was performed non-invasively using a commercially available relocatable mask system as previously described [10]. For planning CT, 3 mm scans of the brain were obtained after administration of an i. v. contrast agent. Treatment planning was performed using Helax TMS (Nucletron, Veenendal, Netherlands) software. For planning target volume (PTV) definition, a margin of 1 to 3 mm around the contrast-enhancing volume was used. Depending on the shape of the PTV, treatment plans were created containing multiple non-coplanar arcs, multiple non-coplanar fixed fields or combinations of both. The treatment plans were normalized to 100% at the isocenter and prescribed to a median isodose of 80% (range 70 to 90%), enclosing the PTV. Details of treatment planning and dose prescription are given in Table 2.

Immediately before the first treatment session, CT simulation was performed as described [10]. Briefly, the isocenter position was verified with regard to bony and parenchymal landmarks and to the contrast enhancing tumor and positioning errors were corrected after attaching the mask system to the treatment table. Treatment was delivered from a Philips SL 75/20 or Siemens Primus linear accelerator (6 MV or 8 MV photons, respectively) equipped with a manual micro-multileaf collimator. Subsequent CT simulations were performed at the discretion of the treating physician depending on the setup error determined at time of the first fraction. HFSRT treatment was performed five days per week. During the HFSRT series, patients were maintained on their previous corticosteroid dose or corticosteroids were started. Daily doses ranged between 32 mg of prednisolone and 40 mg of dexamethasone.

### Statistics

The main endpoint of this study was overall survival (OS) from the time of HFSRT. Kaplan-Meier survival curves were calculated for these intervals considering death as an event and patients alive at last follow-up as censored. Subgroups were compared using the log-rank test. Statistical analysis was performed with Statistica 6.1 software (Tulsa, OK, USA). Overall survival from initial diagnosis and time to tumor progression were considered as secondary endpoints. For the analysis of time to recurrence, neuroradiological diagnoses of follow-up CT and MRI scans were used. Imaging was routinely reviewed by an interdisciplinary tumor board which based its recommendations on these studies.

## Results

HFSRT was well tolerated and no acute neurotoxicity or deterioration in the general health status was observed. Five of 19 patients were reoperated after HFSRT. In three of these, resection was performed for tumor progression on imaging. Histology showed tumor only in two of these cases and predominant tumor with necrosis in one case. In two patients, Ommaya reservoirs were implanted. Thirteen patients received further chemotherapy after HFSRT. Temozolomide was used in six patients, nimustine / teniposide in four and other multi-agent protocols in three.

**Figure 1:**
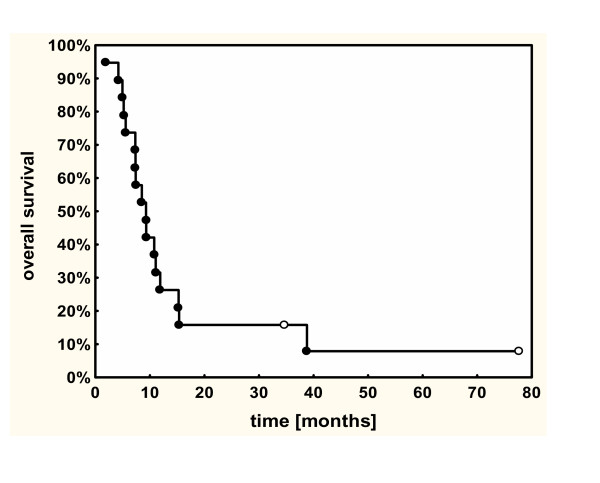
Overall survival of n = 19 patients with recurrent malignant glioma treated with hypofractionated stereotactic radiotherapy (HFSRT), from the time of re-irradiation.

For the whole group of n = 19 patients with recurrent malignant glioma, the median overall survival from the time of HFSRT was 9.3 months (range 1.9 to 77.6+ months; Fig. 1). At the time of analysis, two patients were alive at 34.6 months and 77.6 months. One-year and two-year survival rates were 26% and 16%, respectively. Overall survival from time of HFSRT for subgroups is shown in Table 3.

**Figure 2:**
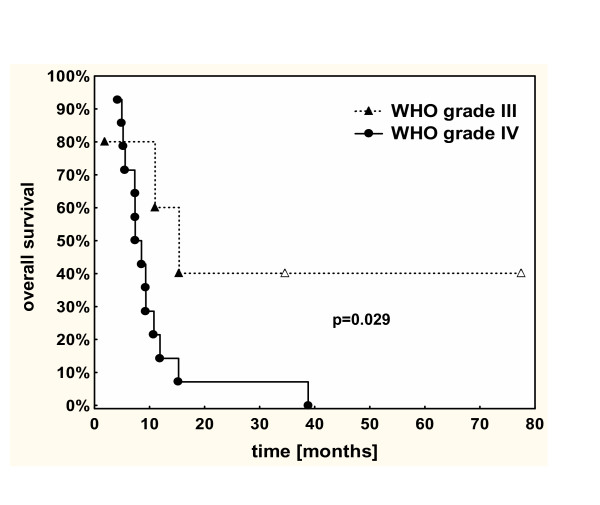
Overall survival from the time of re-irradiation of n = 19 patients with recurrent malignant glioma treated with hypofractionated stereotactic radiotherapy (HFSRT), by WHO grade (most recent histopathology before HFSRT).

**Figure 3:**
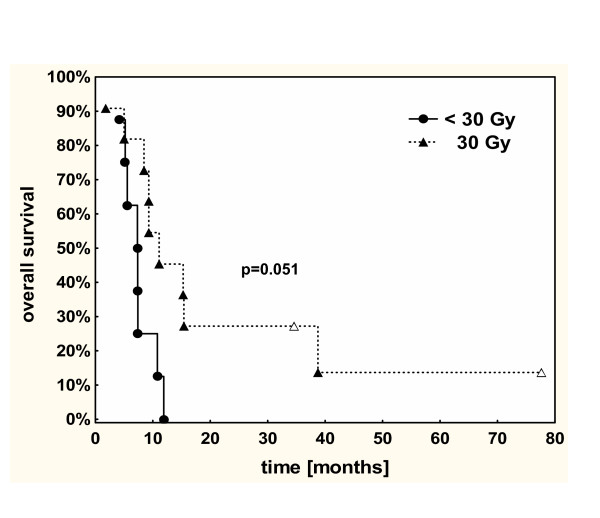
Overall survival from the time of re-irradiation of n = 19 patients with recurrent malignant glioma treated with hypofractionated stereotactic radiotherapy (HFSRT), by total dose.

WHO grading, both that determined at initial diagnosis of glioma and the most recent before HFSRT, had a significant impact on survival. Patients with a most recent histopathology of a grade III glioma had a median overall survival from the time of HFSRT of 15.4 (1.9 to 77.6) months, those with grade IV tumors of 7.9 (4.2 to 38.8) months (p = 0.029, log-rank test; Fig. 2). A trend toward a beneficial effect of higher total doses (30 Gy vs. <30 Gy) on overall survival was observed (p = 0.051; Table 3, Fig. 3). Median overall survival from the first histological diagnosis of glioma (of any grade) was 40.8 months (12.9 to 135 months). Respective median overall survival times from first diagnosis by initial histological grade were 134.7 (64.5 to 135) months for WHO grade II, 57 (30.8 to 119.4+) months for grade III and 26 (12.9 to 40.8) months for grade IV tumors.

Information on follow-up imaging was available in 15 patients. Tumor progression was first diagnosed on MRI in 11 cases and on CT in four cases. The median interval between HFSRT and documented tumor progression was 4.9 months (1.3 to 37.3 months) in the overall group and 7.6 months (3.9 to 37.3 months) and 4.6 months (1.3 to 29.5 months) in the subgroups with a most recent histology of grade III astrocytoma and glioblastoma, respectively.

Survival times from initial diagnosis for the two living patients were 49.7 and 119.4 months. The patient alive at 119.4 months (77.6 months from HFSRT) is a child first treated with HFSRT at the age of eleven for a fourth recurrence of an anaplastic astrocytoma after multiple resections, fractionated radiotherapy and intensive multi-agent chemotherapy. After further adjuvant chemotherapy, this patient recurred 37 months after HFSRT and has since been treated with another resection, a second series of HFSRT (49 months after the first HFSRT) to a volume adjacent to the initial HFSRT region (7 × 5 Gy, 80% isodose) and implantation of BCNU polymers. The most recent MRI in this patient showed no evidence of tumor recurrence. This patient was the only pediatric case included in the present analysis. The patient alive at 49.7 months from initial diagnosis (34.6 months from HFSRT) was first treated for anaplastic astrocytoma at 34 years with biopsy, involved-field radiotherapy and multi-agent chemotherapy. Twelve months after the first radiotherapy series, HFSRT and sequential temozolomide were given for tumor recurrence.

## Discussion

Local recurrence of malignant glioma pretreated with resection, post-operative radiotherapy and frequently with adjuvant chemotherapy is a common problem in clinical practice. Reoperation, re-irradiation and systemic or intratumoral chemotherapy are among the therapeutic options available in this situation. Only the implantation of BCNU polymers at the time of reoperation is supported by randomized trial data, having been shown to be superior to re-resection alone [11]. The benefit of other treatments needs to be evaluated based on phase II data and retrospective analyses which both are prone to bias by patient selection for salvage treatment. A recent large retrospective investigation analyzed the benefit of salvage treatment in patients with glioblastoma multiforme [12]. The authors found that the first, mostly chemotherapeutic, reintervention in this patient group was associated with a doubling in median overall survival from 26 to 61.5 weeks, although selection effects in this series can not be excluded.

Prolongation of survival in recurrent malignant glioma has not been convincingly shown for either re-resection or re-irradiation by interstitial brachytherapy, single-dose radiosurgery or fractionated radiotherapy [13]. Hypofractionated stereotactic radiotherapy (HFSRT) has been proposed as a combination of high-precision treatment with small margins and maximum sparing of normal brain tissue, non-invasive technique and short treatment duration using single fraction doses of 3 Gy to 9 Gy (Table 4).

In the present single-institution series of patients with recurrent malignant glioma, median survival times from the time of HFSRT of 9.3 months (most recent histology grade III 15.4 months, grade IV 7.9 months) were achieved. In published series of HFSRT, applied either as a single modality or in combination with chemotherapy, median overall survival ranged between 7 and 12.7 months (Table 4). Comparability between these series is limited by differences in patient selection and treatment concept. The median total dose of 30 Gy in 5-Gy fractions in the present series is close to the limit of 35 Gy which is thought to be applicable in pre-irradiated patients with acceptable toxicity [4]. However, a total dose of 20 Gy in 4-Gy or 5-Gy fractions, as used in about one third of patients, may be regarded as too low based on recent literature data [4]. Indeed, a trend toward longer survival with total doses of at least 30 Gy was observed (Table 3, Fig. 1C). The finding that no patient in the present series had to be reoperated for symptomatic radiation necrosis also suggests that the dose schedules prescribed were rather conservative. Other potential prognostic factors such as age, Karnofsky performance score or tumor size (planning target volume) were not significant predictors of survival in the present series, possibly due to the limited patient number.

It must be noted that in many patients HFSRT was one element of salvage therapy and further chemotherapy or surgical interventions were performed in 68% and 26% of patients, respectively. In the patients with available imaging information, the median time from HFSRT to tumor progression was approximately five months which may be regarded as an approximation of the lifetime gained by HFSRT in these highly selected patients. In the patient with the longest survival time of 77.6+ months after re-irradiation, a second HFSRT series was performed as well as multiple other treatments, highlighting the benefit of aggressive multi-modality treatment in individual patients. The longest survival time in glioblastoma was 38.8 months from HFSRT, indicating that even in grade IV tumors single patients may survive much longer than expected.

Data from the present series and published reports suggest that efficacy and toxicity are favorable compared to single-dose radiosurgery and the invasive modality of interstitial brachytherapy. While radiosurgery resulted in median overall survival times of 26 to 50 weeks and reoperation rates of 0 to 22%, median overall survival of 47 weeks and reoperation in 41 to 44% (with about 5% of patients showing radionecrosis only) were reported for interstitial brachytherapy [13]. In a recent review of re-irradiation, HFSRT was favored as radiotherapy modality and the following criteria for the application of HFSRT were recommended [14]: good general status (WHO 0–1), at least one year disease-free interval, initial grade II or III histology and maximal tumor diameter 3 cm.

While such criteria may identify patients with the greatest benefit from HFSRT, salvage treatment decisions in recurrent malignant glioma will remain highly individualized. Given the low toxicity of the method, HFSRT may be offered to patients in good general condition with tumor recurrences of limited volume.

## Conclusion

HFSRT with moderate total doses of no more than 30 Gy is safe and leads to similar OS times as more aggressive treatment schemes. In individual patients, HFSRT in combination with other salvage treatment modalities, can be associated with long-term survival.

## Competing interests

The author(s) declare that they have no competing interests.

## Authors' contributions

DV designed the analysis, reviewed patient data, performed statistical analysis and drafted the manuscript.

OK treated the patients, reviewed patient data and revised the manuscript.

KR treated the patients, reviewed patient data and revised the manuscript.

GHV treated the patients, reviewed patient data and revised the manuscript.

KB performed radiotherapy planning for the patients analyzed, reviewed radiotherapy details and revised the manuscript.

MF treated the patients, reviewed patient data and revised the manuscript.

## Pre-publication history

The pre-publication history for this paper can be accessed here:



## Figures and Tables

**Table 1:** Clinical characteristics of n = 19 consecutive patients treated with hypofractionated stereotactic radiotherapy (HFSRT) for recurrent malignant glioma.

median age (range) at time of HFSRT	50 years	(11–74 years)
		
male / female	8 (42%)/11(58%)	
		
median Karnofsky performance score at time of HFSRT (range)	90	(60–90)
		
initial histology		
*astrocytoma II°*	3 (16%)	
*pure anaplastic astrocytoma III°*	4 (21%)	
*anaplastic oligoastrocytoma or oligodendroglioma III°*	3 (16%)	
*glioblastoma multiforme IV°*	9 (47%)	
		
most recent histology before HFSRT		
*anaplastic astrocytoma III°*	5 (26%)	
*glioblastoma multiforme IV°*	14 (74%)	
		
initial surgical procedure		
*resection*	15 (79%)	
*biopsy*	4 (21%)	
		
post-operative radiotherapy		
*1.8–2.0 Gy daily (total dose 54 to 61 Gy)*	12 (63%)	
*3 Gy daily (total dose 45 Gy)*	2 (11%)	
*2 × 1.8 Gy daily (total dose 54 Gy)*	5 (26%)	
		
salvage surgery before HFSRT	12 (63%)	
		
adjuvant or salvage chemotherapy before HFSRT		
*nimustine / teniposide*	12 (63%)	
*temozolomide*	5 (26%)	
*other*	1 (5%)	
		
median interval post-OP radiotherapy to HFSRT (range)	19 months	(3–116 months)

**Table 2:** Treatment details of hypofractionated stereotactic radiotherapy (HFSRT) in n = 19 consecutive patients with recurrent malignant glioma.

Median planning target volume (range)	15 ml	(4–70 ml)
		
Median prescription isodose (range)	80%	(70–90%)
		
Median single dose (range)	5 Gy	(4–10 Gy)
		
Median total dose (range)	30 Gy	(20–30 Gy)
		
Fractionation schedules		
*5 × 4 Gy*	2 (11%)	
*4 × 5 Gy*	5 (26%)	
*6 × 5 Gy*	6 (32%)	
*5 × 6 Gy*	5 (26%)	
*2 × 10 Gy*	1 (5%)	
		
HFSRT technique		
*multiple non-coplanar arcs*	5 (26%)	
*multiple non-coplanar fixed fields*	11 (58%)	
*combination arcs / fixed fields*	3 (16%)	
*median field no. (range)*	5	(3–8)

**Table 3:** Median overall survival from the time of hypofractionated stereotactic radiotherapy (HFSRT) in subgroups of patients with recurrent malignant glioma.

	n	median (range) overall survival (months)	p
WHO grade (initial histopathology)			
*II or III*	10	13.5 (1.9–77.6)	0.036
*IV*	9	7.4 (4.2–10.8)	
			
WHO grade (most recent before HFSRT)			
*III*	5	15.4 (1.9–77.6)	0.029
*IV*	14	7.9 (4.2–38.8)	
			
initial surgical procedure			
*biopsy*	15	9.3 (4.2–77.6)	0.89
*resection*	4	9.2 (1.9–34.6)	
			
chemotherapy before HFSRT			
*yes*	13	9.3 (1.9–77.6)	0.76
*no*	6	8.9 (7.3–15.4)	
			
reoperation before HFSRT			
*yes*	12	8.4 (1.9–77.6)	0.93
*no*	7	9.3 (5.6–34.6)	
			
age at time of HFSRT			
*< 50 years*	11	11.9 (1.9–77.6)	0.14
*> 50 years*	8	8.9 (5.0–11.1)	
			
Karnofsky performance score at time of HFSRT			
*≥ 90*	14	7.9 (4.2–77.6)	0.80
*< 90*	5	11.9 (1.9–15.4)	
			
planning target volume (ml)			
*< 15 ml*	9	9.3 (5.2–38.8)	0.59
*> 15 ml*	10	7.4 (1.9–77.6)	
			
interval first RT series to HFSRT			
*> 20 months*	9	7.4 (1.9–77.6)	0.90
*< 20 months*	10	9.3 (5.6–38.8)	
			
total HFSRT dose			
*30 Gy*	11	11.1 (1.9–77.6)	0.051
*< 30 Gy*	8	7.4 (4.2–11.9)	
			
chemotherapy after HFSRT			
*yes*	13	9.3 (1.9–77.6)	0.27
*no*	6	8.9 (4.2–11.1)	

**Table 4:** Overview of published results on hypofractionated stereotactic radiotherapy (HFSRT) in patients with recurrent malignant glioma (RT: radiotherapy).

**authors**	**patient number**	**single dose (Gy)**	**total dose (Gy)**	**median tumor volume (ml)**	**chemo-therapy**	**overall survival (median)**	**surgery for toxicity**	**prognostic factors**	**comment**
Shepherd et al., 1997 [4]	29 ("high-grade astrocy-toma")	5 (daily)	20–50	24 (3–93)	--	10.7 months	6%	initial low-grade histology associated with longer survival	36% steroid dependent toxicity (increased risk >40 Gy)
Glass et al., 1997 [6]	20 (7 grade III, 13 grade IV)	3.5–6 (twice / week)	35–42	14 (2–122)	cisplatin 40 mg/m^2 ^weekly	12.7 months	15% pre-dominant necrosis on re-operation	--	40% treated within 10 weeks of first RT series for ("potential") progression
Hudes et al., 1999 [5]	19 (glio-blastoma)	3–3.5 (daily)	24–35	13 (1–48)	--	10.5 months	0%	"minor response" on imaging associated with ≥30 Gy and ≤20 ml	recurrent or persistent tumors treated, median interval between completion of RT and HFSRT only 3.1 months
Lederman et al., 2000 [7]	88 (glio-blastoma)	4–9 (weekly)	18–36	32.7 (2–150)	paclitaxel 120 mg/m^2 ^(median) weekly	7 months	8% necrosis only at re-operation	volume ≥30 ml associated with longer survival	median time from diagnosis to HFSRT only 6.5 months
Voynov et al., 2002 [8]	10 (5 WHO grade III, 5 grade IV)	5	30	34.7	--	10.1 months	?	--	stereotactic intensity-modulated radiotherapy (IMRT) used
**present series**	19 (5 WHO grade III, 14 grade IV)	4–10 (daily)	20–30	15 (4–70)	--	9.3 months	0%	WHO grade IV and <30 Gy associated with short survival	median interval post-OP RT to HFSRT 19 months
